# Obituary

**Published:** 1903-10-15

**Authors:** 


### OBITUARY.

### DR. W. C. BARRETT.

Dr. W. C. Barrett, of Buffalo, N. Y., died August 22d, at Nauheim, Germany. at the age of 70 years.

His death occurred from heart failure which has threatened his health for some time; the crisis was precipitated by a violent fall on an icy sidewalk last winter. Since which time he has never been able to do his regular work, and by the advice of his physician he was persuaded, against his wish, to make a visit to the famous spring, of Germany, in hopes that his health might be recovered. In a letter to the editor a few days before he sailed he expressed the wish that he might be spared to see some professional work in which he was intensely interested completed, but he had grave doubts as to his ever returning.

Dr. Barrett was known as widely perhaps as any man in the profession and no man has exceeded his devotion to the general welfare of the profession. He contributed much to the periodic literature, as editor of the *Dental Advertiser & Practitioner* and *Independent Practitioner.*

He also wrote the most practical text-book on Dental Therapeutics in existence. He has also been a constant contributor to literature through the National and other dental societies. As editor of the *Independent Practitioner* he published the papers of Dr. W. D. Miller on dental caries, which introduced Dr. Miller to the dental profession and made him famous. He organized and successfully conducted the dental department of the University of Buffalo, besides giving every year a course of lectures in the Chicago Dental College. He was a lover of the study of comparative anatomy, and not only collected much material of this character, but wrote considerably on the subject. The work of a professional character which last occupied his mind and hand was an effort to establish in foreign countries a proper respect for the United States dental diploma, Doctor of Dental Surgery. For the past five or six years he gave all the time and strength he could command to the work, and he considered that it was so important a work that he was willing to neglect his own personal interests that this work might not fail. In this work he met much opposition and harsh criticism. It remains to be seen if his work shall pass away with his life. He was a man of great earnestness and mental as well as physical alertness. In open debate or controversy he was positive and forceful. To his friends and those who knew him well he was affectionate and ingenuous.

He was such a constant attendant of all the more important dental convocations that he will be greatly missed. Like many other such cases it would seem that his death was untimely, but He who doeth all things well knoweth best, and we mourn our loss in submission.

### DR. HERBERT LEHR.

Dr. Herbert Lehr, of Dwight, Ills., died July 30th of consumption. Dr. Lehr was a graduate of the University of Michigan in 1898. While in college he made all three varsity athletic teams—foot-ball, base-ball and track. He was a fine specimen of physical development and was known in college as "King Lehr." Why such a person should succumb to consumption is a mystery. It is possible that when he left college he quit training too suddenly, and this, with the close confinement of his office, were not conducive to warding off disease.

